# Decoding the Regulatory Mechanism of Astaxanthin on Autophagy: Insights for Anti-Inflammatory Intervention

**DOI:** 10.3390/biom16030477

**Published:** 2026-03-23

**Authors:** Li Feng, Ming Yu, Xiao Ma, Peixi Qin, Yi Zhang

**Affiliations:** Institute of Zoonosis, College of Public Health, Zunyi Medical University, Zunyi 563000, China; 18212147661@163.com (L.F.); 18785363006@163.com (M.Y.); mx23717@163.com (X.M.); qinpeixi@zmu.edu.cn (P.Q.)

**Keywords:** astaxanthin, autophagy, inflammation, regulatory role

## Abstract

Autophagy is a crucial process for cellular self-regulation and renewal. Upon exposure to stress, membrane structures—primarily derived from the endoplasmic reticulum and mitochondria, with contributions from the plasma membrane—drive autophagosome biogenesis. This process begins with the formation of a cup-shaped phagophore, which elongates to sequester cytoplasmic cargo, closes to form an autophagosome, and ultimately fuses with lysosomes to create an autolysosome where degradation and recycling occur. This regulated process plays a vital role in maintaining cellular homeostasis, the pathogenesis of various diseases, and modulation of inflammation. Astaxanthin (AST), a carotenoid produced by microalgae, various microorganisms and marine organisms, possesses a unique chemical structure that endows it with significant biological activities, including potent antioxidant and anti-inflammatory properties. Emerging evidence, primarily from preclinical studies, suggests that AST modulates autophagy by regulating signaling pathways such as Reactive Oxygen Species (ROS)/Mitogen-activated Protein Kinase (MAPK) and interacting with nuclear factor erythroid 2-related factor 2(Nrf2)-mediated antioxidant responses, thereby influencing inflammatory balance. This review systematically elucidates how AST acts as a key “molecular modulator” in animal or cellular models, dynamically regulating autophagy to restore cellular homeostasis and thereby influencing the course and outcome of inflammation. Furthermore, we explore the autophagy-mediated anti-inflammatory effects of AST across different organ systems and discuss its preliminary clinical translational potential and future challenges, aiming to provide a concise and forward-looking roadmap for this promising research field.

## 1. Introduction

Inflammation is a highly complex physiological and pathological process initiated by the body’s response to endogenous or exogenous damaging stimuli [1]. At its core, it is a defensive reaction aimed at isolating and eliminating harmful stimuli while initiating tissue repair and regeneration. This process involves intricate changes in microcirculation, the immune system, and cellular behavior, characterized by the classic signs of redness, swelling, heat, pain, and loss of function [2]. The triggers of inflammation are remarkably diverse, encompassing physical injuries, chemical exposure, and various biological infections, including viral, bacterial, fungal, and protozoan pathogens [3,4]. During the acute phase, immune cells migrate to the injury or infection site through a meticulously orchestrated sequence of events, facilitated by soluble mediators like cytokines and chemokines. However, if the condition persists due to prolonged stimulus exposure or aberrant reactions to self-molecules, inflammation may progress into a chronic stage [5]. Extensive research has confirmed that chronic inflammation is a common underlying factor in many major chronic diseases, including atherosclerosis, metabolic syndrome, neurodegenerative disorders, autoimmune diseases, and various cancers [3,6]. Notably, the initiation, progression, and resolution of inflammation are profoundly influenced by intracellular regulatory mechanisms, among which autophagy plays a particularly critical dual role. While appropriate autophagy helps remove inflammatory triggers and resolve inflammation, dysregulated autophagy can itself act as an inflammatory trigger, contributing to both the initiation of acute inflammation and the sustained activation of immune cells and pro-inflammatory signaling that drives chronic inflammation [7].

Autophagy is a highly conserved intracellular degradation and recycling system in eukaryotic cells, playing a critical role as a “cleaner” and “recycling center” in maintaining cellular homeostasis. Its primary functions include clearing misfolded or aggregated proteins, degrading dysfunctional or excess organelles (such as mitochondria and endoplasmic reticulum), and eliminating intracellular pathogens, thereby providing nutrients and maintaining internal stability. Depending on how substrates are delivered to the lysosome, autophagy is classified into three main types: microautophagy, macroautophagy, and chaperone-mediated autophagy. Among these, macroautophagy is the most extensively studied and has the broadest physiological significance [8]. The initiation and execution of autophagy are precisely regulated processes. Under stress signals such as oxidative stress, the mammalian target of rapamycin (mTOR) kinase activity is suppressed, while the AMP-activated protein kinase (AMPK) pathway is activated, both of which act on the UNC-51-like kinase 1(ULK1)/2-ATG13-FIP200 complex to initiate autophagy [9,10]. Subsequently, c-Jun N-terminal kinase 1 (JNK1) phosphorylates endoplasmic reticulum-localized BCL-2 at residues T69, S70, and S87, leading to the dissociation of BCL2 interacting protein (Beclin1) [11]. Liberated Beclin1 then activates the Beclin-1-Vps34 (class III phosphatidylinositol 3-kinase, PI3K) complex, promoting the production of phosphatidylinositol 3-phosphate (PI3P). PI3P recruits effector proteins such as WIPI2, thereby facilitating the recruitment of the ATG12-ATG5-ATG16L1 complex and the lipidation of Microtubule-associated Protein 1 Light Chain 3(LC3), driving phagophore elongation [12]. Thereafter, two ubiquitin-like conjugation systems—the ATG12-ATG5-ATG16L1 complex and the lipidation system converting LC3-I to phosphatidylethanolamine-conjugated LC3-II—cooperate to drive the extension and closure of the phagophore, ultimately forming an autophagosome that sequesters cytoplasmic contents [13]. Mature autophagosomes fuse with lysosomes through membrane fusion proteins including syntaxin-17 (STX17), synaptosomal-associated protein 29 (SNAP29), and vesicle-associated membrane protein 8 (VAMP8), forming autolysosomes in which the contents are thoroughly degraded by various hydrolytic enzymes, and the degradation products are recycled back into the cytoplasm [14]. Autophagy serves as a bridge that links cellular homeostasis with systemic immune responses. By directly eliminating pathogens, processing antigens, suppressing excessive inflammation, and supporting immune cell survival, it fine-tunes both the intensity and the tolerance of the immune system. While the immune system defends the body by initiating inflammation—a vital mechanism for pathogen clearance and tissue repair—autophagy and inflammation engage in a complex and bidirectional regulatory interaction [15,16]. On one hand, at baseline, autophagy in alveolar macrophages effectively suppresses spontaneous pulmonary inflammation [17]. On the other hand, uncontrolled autophagy—whether due to insufficient function or excessive activation—can promote cell death (such as apoptosis and necroptosis) and release damage-associated molecular patterns, thereby exacerbating the inflammatory response and creating a vicious cycle [18]. This ‘double-edged sword’ property highlights the precise regulation of autophagy as a promising mechanistic target for intervening in inflammation-related diseases.

Astaxanthin (AST) is a naturally occurring lipid-soluble red-orange pigment (chemical name: 3, 3′-dihydroxy-β, β′-carotene-4,4′-dione) belonging to the xanthophyll carotenoid family [4,19], widely used in dietary supplements for health or nutritional support [20]. It is mainly synthesized by microalgae, such as *Haematococcus pluvialis*, in marine environments and accumulates in marine organisms like shrimp, crabs, and salmon through the food chain [21]. Its unique molecular structure—characterized by two ends containing a hydroxyl and keto group and a polyene long chain composed of 13 conjugated double bonds—forms the chemical basis for its exceptional biological activities [22,23]. Compared to natural compounds such as vitamin D3, trehalose, and spermine, AST provides broader membrane protection, stronger antioxidant activity, enhanced stability, and more precise bidirectional regulation of autophagy. Additionally, it holds FDA GRAS (Generally Recognized as Safe) certification, attributed to its unique molecular structure [24,25,26,27,28]. Recent cutting-edge research has further revealed that AST can profoundly influence the process of cellular autophagy. Rather than merely activating or inhibiting autophagy, it acts as a “regulator,” finely tuning autophagy in response to the cellular microenvironment or immune system status. For instance, under oxidative stress conditions, AST can restore impaired autophagy by inducing mitophagy [29] or directly regulating the expression and function of key autophagic proteins such as Beclin-1, LC3-II, and Sequestosome-1 (SQSTM1/p62), thus providing cellular protection [30]. Notably, AST regulates autophagy and activity to influence inflammatory responses, thereby alleviating disease progression and reducing organ damage. Examples include enhancing podocyte autophagy and inhibiting mesangial cell pathological crosstalk to alleviate diabetic kidney injury [31], or alleviating NLRP3-mediated inflammation to improve Age-Related Macular Degeneration (AMD) [32]. At both cellular and organ levels, AST can ameliorate inflammatory damage induced by autophagy.

Given these finding, elucidating the regulatory role of AST in the complex interplay between autophagy and inflammation is critical not only for understanding the biological activity of this natural compound, but also for developing novel intervention strategies for inflammatory diseases. Although previous reviews have summarized the anti-inflammatory properties of AST, the specific mechanisms by which it modulates autophagy to influence inflammatory outcomes remain to be systematically synthesized. This review provides a conceptual framework that integrates current preclinical evidence, critically evaluates how AST regulates autophagy across different organ systems in animal and cellular models of inflammation, and assesses the translational gap between these promising preclinical findings and the requirement for future clinical validation. By delineating this research landscape, we aim to offer a forward-looking roadmap that identifies key unanswered questions and guides future research directions in this field.

## 2. Methodology

We conducted a systematic review to comprehensively analyze and summarize studies exploring the regulatory mechanism of AST on autophagy in the context of anti-inflammatory intervention. The following academic databases comprising a broad, authoritative literature base, including PubMed, EMBASE, and Web of Science have been explored. The search terms were meticulously formulated as “astaxanthin” and its synonyms “AST” and “3,3′-dihydroxy-β,β-carotene-4,4′-dione”. The related terms regarding autophagy were formulated as “autophagy”, “autophagic”, “macroautophagy”, “mitophagy”, and “autophagosome”, while inflammatory-related terms included “anti-inflammatory”, “inflammation”, “inflammatory”, and “cytokines”. To select the relevant studies, the final search formula was created as follows: (“astaxanthin” OR “AST” OR “3,3′-dihydroxy-β,β-carotene-4,4′-dione”) AND (“autophagy” OR “autophagic” OR “macroautophagy” OR “mitophagy” OR “autophagosome”) AND (“anti-inflammatory” OR “inflammation” OR “inflammatory” OR “cytokines”).

**Figure 1:**
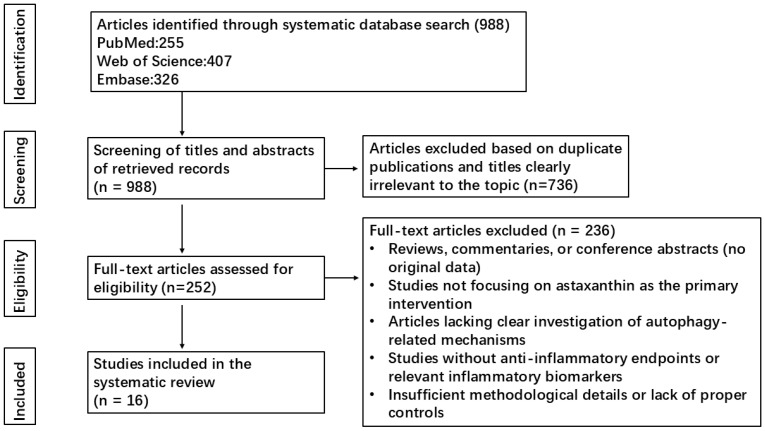
The literature search methodology.

Our initial search yielded 988 publications, including 255 from PubMed, 407 from EMBASE, and 326 from Web of Science. Subsequently, 736 duplicate articles and those whose titles were considered irrelevant to our topic were eliminated following meticulous screening of titles and abstracts. The remaining 252 articles were thoroughly evaluated, and 236 studies were ultimately excluded due to the following criteria: (i) reviews, commentaries, or conference abstracts without original experimental data; (ii) studies not focusing on AST as the primary intervention; (iii) articles lacking clear investigation of autophagy-related mechanisms; (iv) studies without anti-inflammatory endpoints or relevant inflammatory biomarkers; and (v) insufficient methodological details or lack of proper controls. Finally, 16 studies were included based on their relevance to AST-mediated autophagy regulation in anti-inflammatory intervention and the scientific rigor of their designs (Figure 1).

## 3. Regulatory Effects of AST on Autophagy Signaling Pathways

**Figure 2:**
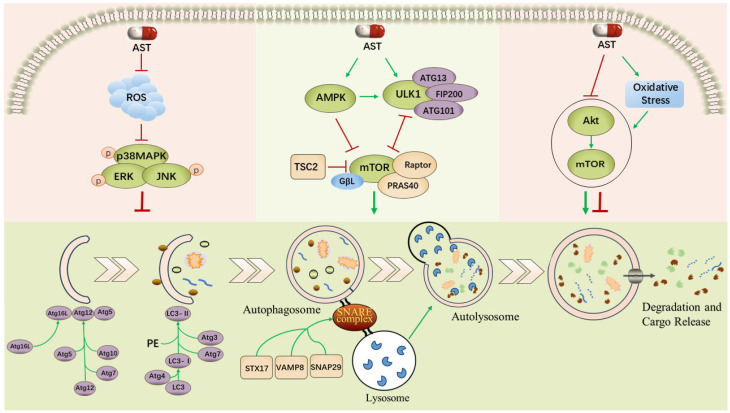
The Regulatory Network of Autophagy by AST.

Autophagy is a key process to maintain cell homeostasis, and its regulation involves the precise integration of multiple signaling pathways. Studies have shown that moderate autophagy activation has a protective effect on cells; however its imbalance is closely related to the pathogenesis of many diseases. Notably, we found that AST can regulate the autophagy process through multiple pathways, including Reactive Oxygen Species (ROS)/Mitogen-activated Protein Kinase (MAPK), AMPK-mTOR-ULK1, protein kinase B (PKB/Akt)/mTOR, nuclear factor erythroid 2-related factor 2 (Nrf2), as well as via the NLRP3 inflammasome (Figure 2).

The schematic illustrates that AST modulates cellular autophagy through multiple signaling pathways. Under stress stimulation, AST reduces ROS generation, thereby inhibiting the phosphorylation levels of MAPK family members (p38 MAPK, JNK, and ERK) and ultimately suppressing autophagy. Additionally, AST promotes autophagosome formation by activating AMPK to directly inhibit mTOR complex assembly; it further enhances autophagy by alleviating the inhibitory effect of mTOR on the ULK1 complex. Notably, in the Akt/mTOR signaling pathway, Jia et al. demonstrated that AST promotes hepatic autophagy by inhibiting Akt/mTOR, whereas Yan et al. reported that AST suppresses mitophagy through activation of Akt/mTOR, illustrating the bidirectional regulatory effect of AST on autophagy. In this figure, red “┬” arrows denote reduced levels of autophagy-related markers (e.g., LC3-II, Beclin1) or increased p62 accumulation, indicating inhibition of autophagic activity. Green “↑” arrows denote elevated levels of such markers or decreased p62 accumulation, indicating promotion of autophagic activity. The ROS/MAPK signaling pathway is adapted from Li et al. [33]. and the process of autophagosome formation and maturation is adapted from Yu et al. [19]. The remaining content is original.

### 3.1. Regulation of Autophagy by AST Through ROS/MAPK Family Signaling Pathway

ROS are oxygen-containing active molecules produced by cellular metabolism. Excessive ROS can cause oxidative damage and significantly affect normal bodily functions. Studies have shown that as a key molecule sensitive to oxidative stress, AST can eliminate ROS, alleviate oxidative stress, prevent mitochondrial autophagy overload, and thereby maintain the integrity and function of mitochondria [34]. The MAPK family is a core signaling hub for cellular stress and energy state perception, including subtypes such as p38 MAPK, extracellular signal-regulated kinase (ERK), and JNK, whose activities are dynamically regulated by phosphorylation and dephosphorylation [35,36,37]. Notably, AST exerts a crucial regulatory effect on autophagy through the ROS/MAPK signaling pathway. For instance, it has been confirmed in the liver ischemia–reperfusion (IR) injury model that AST attenuated ROS and cytokine mediated phosphorylation of p38 MAPK, JNK, and ERK, thereby reducing apoptosis and autophagy [33].

In addition, the JNK pathway is a crucial branch in the MAPK cascade that senses stress. It also plays an important role in regulating autophagy. In a model of autoimmune hepatitis, AST has been shown to alleviate cellular autophagy by blocking JNK phosphorylation, which in turn reduces ROS and suppresses inflammatory factors [38]. Collectively, this evidence suggests that AST effectively scavenges ROS and inhibits the phosphorylation of key MAPK family members (p38/ERK/JNK), thereby modulating autophagic activity to ultimately support maintenance of cellular homeostasis.

### 3.2. Regulation of Autophagy by AST Through the AMPK-mTOR-ULK1 Signaling Pathway

AMPK is a heterotrimeric kinase that plays a crucial role in maintaining mitochondrial health and promoting mitochondrial autophagy [39]. It can promote autophagy by activating the AMPK pathway, thereby inhibiting the occurrence of related diseases [40]. For instance, in the model of *Helicobacter pylori (H. pylori)*-infected human gastric epithelial cell line AGS (adenocarcinoma gastric), AST was shown to activate AMPK while suppressing its downstream targets, mTOR and Akt, as evidenced by increased p-AMPK and reduced p-mTOR/p-Akt levels. This regulatory effect promotes autophagic induction, characterized by an elevated LC3B-II/LC3B-I ratio, decreased p62 expression, and a significant increase in LC3B puncta [40]. The mTOR protein is a central regulator of cell growth and metabolism, and plays a key role in the negative regulation of autophagy. Studies have found that AMPK can inhibit the activity of mTOR to relieve the inhibition of the autophagy initiation complex ULK1 by mTOR, thereby positively regulating autophagy [9,41]. AST plays important regulatory roles involving this process. For example, For example, in SH-SY5Y human neuroblastoma cells, AST-DHA activates the AMPK/ULK1 pathway and inhibits mTOR-mediated ULK1 phosphorylation, thereby increasing autolysosome formation and the autolysosome/autophagosome ratio, which is consistent with enhanced autophagosome-lysosome fusion and correlates with reduced Aβ_1–42_ accumulation and attenuated Tau protein phosphorylation [41]. Another study demonstrated that AST directly targets mTOR to regulate cellular autophagy. In a lipopolysaccharide (LPS)-induced ex vivo hippocampal inflammatory model, AST exposure induces autophagy in primary porcine brain capillary endothelial cells, thereby promoting the clearance of misfolded proteins, as evidenced by increased levels of LC3B-II and decreased phosphorylation levels of mTOR and S6 ribosomal protein [42]. Collectively, these findings indicate that AST can exert a crucial influence on autophagy by regulating the AMPK-mTOR-ULK1 signaling network.

### 3.3. Regulation of Autophagy by AST Through the Akt/mTOR Signaling Pathway

The Akt/mTOR pathway plays a crucial role in autophagy. Inhibiting this pathway can rapidly and effectively initiate autophagy, providing a key target for the intervention of various diseases. Notably, AST can regulate autophagy through the Akt/mTOR axis. For instance, in a mouse model of high-fat diet-induced metabolic inflammation, AST alleviated hepatic inflammation by activating PPARα and inhibiting the Akt-mTOR pathway, while reducing TNF-α and IL-6 levels and promoting hepatic autophagy [43]. In addition, in the H_2_O_2_-induced oxidative damage model of SH-SY5Y cells, AST can inhibit mitophagy by promoting Akt/mTOR signaling, thereby suppressing H_2_O_2_-induced cytotoxicity and reducing oxidative damage in neurons [44]. Thus, AST bidirectionally regulates the Akt/mTOR-autophagy axis in a context-dependent manner at both cellular and animal levels, providing multiple strategies for precise autophagy-targeted interventions.

### 3.4. Regulation of Autophagy by AST Through Other Signaling Pathways

Nrf2 is a key regulator of cellular antioxidant responses, and recent studies have shown that it can also be involved in the autophagy process [45]. AST is an effective activator of the Nrf2 pathway. For example, in models of acute pancreatitis (AP), AST attenuated inflammatory injury by activating Nrf2, which improved defective autophagic processes and reduced the release of pro-inflammatory cytokines (TNFα, IL6, and IL-1β), thereby alleviating the pathological progression of the disease [46].

The NLRP3 inflammasome and the Nuclear Factor kappa-light-chain-enhancer of activated B cells (NF-κB) signaling pathway are critical components of innate immunity, with their activation closely linked to the autophagy process. In a deoxynivalenol (DON)-induced immune injury model, AST activates the PINK1-Parkin-mediated mitophagy pathway to eliminate damaged mitochondria and mitochondrial reactive oxygen species (mtROS). This action suppresses the mtROS-NF-κB-NLRP3 signaling axis, effectively alleviating DON-induced immunotoxicity, pyroptosis, and inflammatory responses in lymphocytes [47]. Therefore, AST enhances redox status and autophagic function by activating the Nrf2 pathway while simultaneously suppressing the mtROS-NF-κB-NLRP3 signaling axis through PINK1-Parkin-mediated mitophagy. This dual regulation of autophagy contributes to the attenuation of inflammatory damage.

In summary, AST exhibits characteristics as a “multi-pathway regulator”. By activating AMPK/ULK1 and inhibiting the mTOR signaling pathway—coordinating with Akt/mTOR—it senses cellular energy status and initiates protective autophagy. Concurrently, it effectively disrupts harmful signaling cascades triggered by oxidative stress and inflammatory factors through suppressing the over-activation of MAPK family members (e.g., p38, JNK, ERK). Furthermore, as a potent activator of the Nrf2 pathway, AST not only enhances overall cellular antioxidant capacity and coordinates autophagosome formation, but also attenuates inflammation by suppressing NLRP3 inflammasome activation via the NF-κB axis. Together, these actions establish a collaborative protective network across multiple organ disease models.

## 4. AST’s Impact on Inflammation Through Autophagy Regulation: Research and Applications

**Figure 3:**
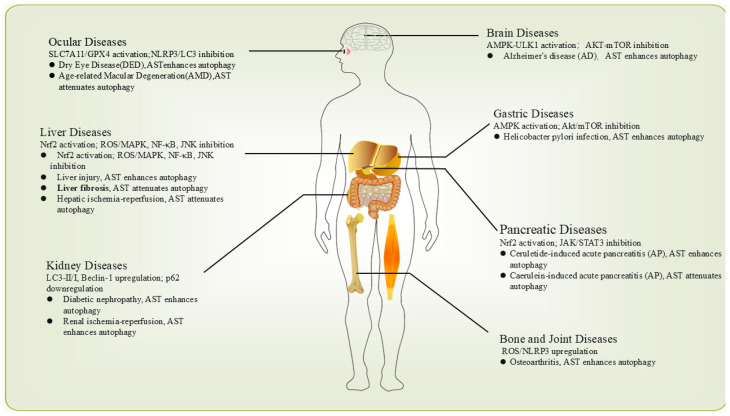
AST Modulates Autophagy to Alleviate Organ-Specific Inflammatory Diseases.

Autophagy is a critical cellular homeostasis mechanism, and its dysregulation is closely associated with the pathogenesis of various inflammatory diseases [48]. As discussed in the preceding section, AST is a multifaceted natural compound that fine-tunes autophagic activity by modulating key signaling pathways, including AMPK/ULK1/mTOR, ROS/MAPK, Akt/mTOR, Nrf2, and the NLRP3 inflammasome. This dual regulatory capability forms the foundation of its potent antioxidant and anti-inflammatory effects at both cellular and molecular levels. Building on these insights, this chapter delves into the protective effects of AST in organ systems commonly affected by inflammatory pathologies. Particular attention is given to its roles in the joints, eyes, brain, liver, digestive tract, and urinary system, with a focus on synthesizing recent research advances that shed light on AST’s ability to modulate autophagy and influence disease progression (Figure 3). Additionally, we discuss its potential as a nutritional strategy and its possible adjunctive role in supportive care, aiming to inform and guide future research directions.

This schematic illustrates how AST regulates autophagy to mitigate inflammation across key human organs. In the eye, AST enhances autophagy via SLC7A11/GPX4 activation to alleviate dry eye disease (DED) and inhibits NLRP3/LC3 to attenuate age-related macular degeneration (AMD). For liver diseases, AST enhances autophagy (e.g., liver injury) or attenuates excessive autophagy (e.g., liver fibrosis, hepatic ischemia–reperfusion (IR)) by modulating Nrf2, ROS/MAPK, NF-κB, and JNK pathways. In kidney diseases such as diabetic nephropathy and renal IR, AST enhances autophagy by upregulating LC3-II/I and Beclin-1 while downregulating p62. For bone and joint diseases like osteoarthritis, AST enhances autophagy by suppressing ROS/NLRP3 upregulation. In brain diseases including Alzheimer’s disease (AD) and subarachnoid hemorrhage, AST enhances autophagy via AMPK-ULK1 activation and AKT-mTOR inhibition. In gastric diseases such as *H. pylori* infection, AST enhances autophagy by AMPK activation and Akt/mTOR inhibition. For pancreatic diseases like cerulein- or caerulein-induced acute pancreatitis (AP), AST enhances or attenuates autophagy by modulating Nrf2 and JAK/STAT3 pathways. This figure is original and was created by the authors.

### 4.1. Application of AST in Joint Inflammation

Osteoarthritis (OA) is a chronic degenerative disease characterized by cartilage degeneration and synovitis, closely related to age, obesity, and chronic low-grade inflammation [49,50]. Inflammation, particularly the imbalance of synovial macrophages (M1/M2), is a critical factor driving the progression of OA [51]. Recent studies have shown that AST can alleviate OA symptoms through nanotechnology. In an ACLT-induced mouse model, intra-articular injection of ROS-responsive nanoparticles containing AST (NP@PolyRHAPM) significantly reduced knee width and claw circumference, decreased OARSI scores, and protected articular cartilage through regulating autophagy [52] (Table 1). Given that conventional treatments such as non-steroidal anti-inflammatory drugs primarily alleviate symptoms and carry long-term side effects [53], AST has garnered attention as a dietary supplement candidate worthy of further investigation for its potential role in supporting joint health.

### 4.2. Application of AST in Eye Inflammation

Dry Eye Disease (DED): DED is a common ocular disorder whose pathogenesis involves the activation of oxidative stress pathways in corneal epithelial cells, leading to cell necrosis and autophagy and ultimately contributing to ocular inflammation [59]. Studies have shown that AST exerts a protective effect against DED. In a scopolamine-induced mouse model, topical AST intervention significantly reduced corneal fluorescein staining scores, rescued ocular surface defects, restored corneal epithelial integrity, and mitigated mitochondrial ultrastructural damage. These eye-specific protective effects were mediated through AST activation of the SLC7A11/GPX4 axis to inhibit ferroptosis and restore healthy autophagy [54]. Therefore, AST may aid in the prevention of DED by maintaining autophagic function, suppressing the release of inflammatory factors, and mitigating ocular surface inflammation.

Age-Related Macular Degeneration (AMD): In dry AMD, the excessive activation of the NLRP3 inflammasome in retinal pigment epithelial (RPE) cells and autophagy imbalance are central pathological aspects [32]. Research indicates that AST reduces abnormal autophagy (reflected by changes in the LC3-II/LC3-I ratio) induced by sodium iodate (NaIO_3_) and alleviates NLRP3-mediated inflammation, displaying effects comparable to the specific NLRP3 inhibitor CY-09 [32].

Collectively, these studies demonstrate that AST has health-promoting potential for intervening in ocular inflammation by regulating autophagy homeostasis and inflammasome activity.

### 4.3. Application of AST in Liver Injury

In liver injury, dysfunctional autophagy and excessive inflammatory responses form a lethally synergistic relationship. Impaired autophagy leads to the accumulation of inflammatory signaling molecules and strongly activates inflammatory pathways; conversely, the inflammatory environment disrupts normal autophagic processes. The core protective mechanism of AST lies in restoring the cell’s self-cleaning capacity (autophagy/mitophagy), thereby curbing the source of inflammation upstream while directly modulating inflammatory signaling pathways. Ultimately, this stabilizes hepatocyte metabolism and the internal environment, achieving multi-targeted hepatoprotective effects.

Acetaminophen-Induced Liver Injury (AILI): Studies have shown that a hollow mesoporous silica nanoparticle loaded with AST (HMSN@ASX) has been developed to enhance the aqueous solubility of AST and enable its targeted delivery to the mouse liver. AST prevents AILI by activating the Nrf2/heme oxygenase-1 (HO-1) pathway to enhance autophagy and reduce ferroptosis (as manifested by increased expression of autophagy-related genes LC3A and LC3B, elevated LC3B/LC3A protein ratio, and decreased p62 levels) [55].

Liver Fibrosis: Liver fibrosis is primarily caused by chronic liver injury, and the activation of hepatic stellate cells (HSCs) is a key process in its development. Shen et al. confirmed that AST (80 mg/kg) can alleviate liver fibrosis by down-regulating autophagy levels, thereby reducing energy production in HSCs [56]. Additionally, AST exerts immunomodulatory effects by inhibiting the NF-κB signaling pathway, thereby reducing the expression of pro-inflammatory cytokines such as Transforming Growth Factor-beta 1 (TGF-β1) and suppressing macrophage activation, which further attenuates the inflammatory response and HSC activation in the fibrotic liver [56,60]. Autophagy regulation is one of the key mechanisms underlying the anti-fibrotic effects of AST. Although AST does not directly inhibit HBV or HCV replication, it effectively suppresses virus-induced liver fibrosis by blocking the TGF-β1/Smad3 signaling pathway [61]. Therefore, the regulatory role of AST in virus-induced autophagy during HBV/HCV infection (e.g., through mechanisms such as HBx-p7-Beclin-1) warrants further investigation [62].

Autoimmune Hepatitis and Ischemia–Reperfusion Injury: In hepatic IR injury, AST pre-treatment inhibited hepatocyte apoptosis and autophagy, thereby dose-dependently attenuating serum ALT/AST elevations and hepatocellular necrosis evident on HE staining, ultimately preserving liver architecture and function [33]. Moreover, in ConA-induced autoimmune hepatitis, AST attenuates immune-mediated liver injury by suppressing aberrant JNK signaling, which decreases the expression of autophagy markers LC3 and Beclin-1 and reduces autophagosome formation in hepatic stellate cells [38].

### 4.4. Application of AST in Injury of the Digestive and Urogenital Systems

Digestive System: The gastrointestinal mucosa is chronically exposed to oxidative stress and an inflammatory milieu such as that induced by *H. pylori* infection, which constitutes a risk factor for ulceration, colitis, and even carcinogenesis [40,63]. Studies have shown that AST increases autophagy in *H. pylori*-infected AGS cells by activating the AMPK signaling axis while concurrently inhibiting the Akt and mTOR pathways. Specifically, this is manifested by elevated LC3B-II/LC3B-I ratio, reduced p62 levels, and increased numbers of LC3B puncta and acidic vesicular organelles (AO-positive cells) in *H. pylori*-stimulated cells [40]. In AP, AST pre-treatment dose-dependently reduced serum amylase and lipase levels, attenuated pancreatic histological damage (edema, acinar cell necrosis, and inflammatory infiltration), and decreased pancreatic myeloperoxidase activity and proinflammatory cytokine production (TNF-α, IL-6, IL-1β). Mechanistically, AST inhibited acinar cell ferroptosis by restoring mitochondrial morphology, reducing lipid peroxidation products (MDA, 4HNE), and promoting Slc7a11 membrane localization, while also suppressing excessive autophagy and apoptosis in pancreatic tissues [46,57].

Urinary System: In models of diabetic nephropathy, AST alleviates renal inflammation through multi-target synergistic effects: it downregulates the expression of TGF-β2/VEGF-B and inhibits α-SMA/collagen IV, thereby conferring direct renal protection. Concurrently, AST upregulates the LC3-II/I ratio and reduces p62 levels, suggesting the restoration of autophagic activity in podocytes and a subsequent reduction in the release of pathogenic factors. Furthermore, by blocking high glucose-induced VEGF-B/TGF-β2 signaling crosstalk between podocytes and mesangial cells, AST achieves comprehensive renal protection [31]. In kidney IR injury, high-dose AST (25 mg/kg) preserved renal function, as evidenced by reduced serum blood urea nitrogen (BUN) and creatinine levels. It also ameliorated tubular degeneration, including necrosis and dilatation, as well as interstitial inflammatory infiltration in the cortex and medulla. Mechanistically, AST activated autophagy specifically in renal tubular epithelial cells, characterized by increased immunoreactivity of Beclin-1 and LC3β, along with decreased expression of p62. This activation provides renal protection against oxidative damage [30]. As mentioned above, AST primarily improves kidney tissue damage by enhancing protective autophagy. However, it cannot be overlooked that long-term high-dose use of AST may impose a metabolic burden on the liver and kidneys [64]; therefore, further practical exploration is still needed regarding dosage control and duration of use in the future.

Testicular torsion: Testicular torsion is a significant clinical condition that leads to permanent ischemic damage and subsequent loss of function in testicular tissue. AST can regulate the expression of the key autophagy protein Beclin-1, activate autophagy and simultaneously inhibit the Cysteine-aspartic Protease 3(caspase-3)-mediated apoptotic pathway, thereby further protecting the structure and function of the seminiferous epithelium and significantly alleviating testicular IR injury [58]. However, studies have shown that in the reproductive system, the bidirectional regulation of hormones by AST complicates its effects: on one hand, it promotes testosterone synthesis in the testes, while on the other hand, it inhibits testosterone and dihydrotestosterone (DHT) levels in the prostate [65]. Preclinical studies suggest that AST may exert beneficial effects on testicular ischemia–reperfusion injury, indicating a potential therapeutic role worthy of further investigation [66], However, while AST is commercially available as a dietary supplement, its clinical efficacy and safety in this specific context remain to be established, and its potential adverse effects should not be overlooked.

### 4.5. Application of AST in Neuroinflammation

AST exhibits neuroprotective effects in various models of neuroinflammation and injury. In Alzheimer’s disease (AD) models, AST enhances autophagy while reducing β-amyloid (Aβ) deposition and Tau hyperphosphorylation. It achieves this by activating the AMPK-ULK1 pathway and inhibiting the Akt-mTOR axis, ultimately alleviating blood–brain barrier dysfunction and neuroinflammation [41,42]. In the H_2_O_2_-induced SH-SY5Y neuronal cell injury model, AST alleviates neuronal damage and maintains mitochondrial quality and function by activating the Akt/mTOR signaling pathway and suppressing PINK1/Parkin-mediated excessive mitophagy (as evidenced by downregulated expression levels of key mitophagy-related proteins including Beclin1, LC3-II, PINK1, and Parkin) [44].

In summary, extensive research demonstrates that AST exerts broad and effective protective effects in the prevention and intervention of various inflammation-related diseases. On the basis of this evidence, the core mechanism of AST lies in its ability to bidirectionally and dynamically modulate cellular autophagy in a context-dependent manner across different organs and pathological conditions. Rather than simply turning autophagy “on” or “off,” AST exerts a comprehensive regulatory effect on pivotal signaling hubs—encompassing energy-sensing, stress-response, and inflammation-related pathways—thereby restoring impaired autophagic homeostasis across diverse pathological conditions. Generally, in osteoarthritis, AST protects cartilage by regulating the autophagy-inflammation network; in DED and AMD, it alleviates ocular surface and retinal inflammatory damage by enhancing protective autophagy or suppressing aberrant autophagy; in liver injury models, it interrupts the vicious cycle of inflammation by bidirectionally regulating hepatocyte autophagy; and in neurodegenerative diseases, it exerts neuroprotective effects by clearing toxic protein aggregates and improving brain function through autophagy modulation.

Thus, AST’s role extends beyond traditional antioxidant activity. As both an “autophagy fine-tuner” and a “multi-pathway network modulator,” its potential application in chronic inflammatory diseases is primarily based on in vitro cell experiments and animal model studies. However, it must be pointed out that effective dosages, safe formulations, and clinical outcome data for AST in humans are currently severely lacking, and a significant gap exists between the existing evidence and clinical translation. Future research is urgently needed to bridge this gap: first, optimizing the bioavailability of AST and developing disease-specific delivery strategies, and subsequently conducting rigorously designed randomized controlled trials to validate its long-term safety and efficacy in humans.

## 5. Advancing AST: From Molecular Insights to Clinical Applications

AST has emerged as a compound of interest for its role in modulating autophagy, a process implicated in the pathogenesis of various chronic diseases. However, its development as a reliable therapeutic agent faces several critical challenges, underscoring the need for systematic advancements in future research. First, while current studies provide evidence of AST’s potential, they largely remain focused on phenotypic associations. The direct upstream molecular targets of AST—such as specific membrane receptors or intracellular sensor proteins—have yet to be clearly identified. To enhance the translational rigor of this field, future studies must prioritize the methodological diversification of experimental models. Notably, as a significant portion of current in vitro evidence is derived from single cell lines, it is imperative that forthcoming research validates these findings across multiple relevant cell types or primary cell lines. Such cross-model validation is essential to account for cellular heterogeneity and to confirm the reproducibility of AST’s observed biological effects. Furthermore, by integrating advanced techniques in chemical biology and proteomics, researchers can more robustly pinpoint the primary molecular switches—such as the orchestrated modulation of AMPK, mTOR, and Nrf2—that drive AST’s synergistic protection across diverse biological contexts. This will not only deepen our understanding of AST as a “multi-pathway network modulator” but also provide a foundation for structure-based drug optimization. Moreover, there remains a substantial translational gap between preclinical findings and human applications. Current evidence is predominantly derived from cell and animal models, where the doses, routes of administration, and physiological contexts differ significantly from those in humans. To bridge this gap, systematic pharmacokinetic studies are essential to elucidate the absorption, distribution, metabolism, and excretion (ADME) profiles of various AST formulations, including esterified derivatives and nano-formulations, in humans. Identifying optimal dose–response relationships and therapeutic windows for specific diseases—such as neurodegenerative diseases—while rigorously evaluating the long-term safety of AST use, will be critical for its clinical translation.

Addressing AST’s inherent limitations in bioavailability and stability is another key priority. Its low solubility and instability hinder effective accumulation at disease sites. Future research should move beyond conventional encapsulation methods and focus on developing advanced, intelligent delivery systems. For instance, nanocarriers responsive to pathological cues such as elevated reactive ROS or disease-specific enzymes could enable targeted delivery to inflammation sites, atherosclerotic plaques, or tumor microenvironments. Overcoming barriers like the blood–brain barrier and the retinal barrier will require bioinspired or functionalized delivery platforms to ensure efficient transport to the central nervous system and the eye. Additionally, co-delivery strategies that combine AST with other autophagy modulators, such as rapamycin, could enhance therapeutic synergy.

Prospectively, translating preclinical findings on AST into clinical applications will require a comprehensive and interdisciplinary approach that bridges basic and clinical research. This includes uncovering its molecular targets, developing innovative delivery systems, and designing well-controlled clinical trials to evaluate its efficacy and safety. By integrating these efforts, AST may ultimately be positioned as an evidence-based nutritional strategy for supporting health and managing disease risk through autophagy modulation.

## 6. Conclusions

AST is a natural compound that regulates autophagy through multiple signaling pathways, including AMPK-mTOR-ULK1, MAPK, Akt/mTOR, and Nrf2. By fine-tuning autophagic activity, AST helps maintain cellular balance and reduces inflammation in various diseases, such as osteoarthritis, liver injury, kidney damage, and neuroinflammation. Notably, AST does not simply activate or inhibit autophagy; rather, its regulatory direction is context-dependent, balancing protective and excessive autophagic responses across different diseases. Therefore, we propose that AST is a natural compound capable of bidirectionally regulating autophagy to influence inflammatory responses and maintain homeostasis, with the potential for multi-target and multi-organ clinical applications. Nevertheless, the path to clinical application is hindered by unresolved issues—limited bioavailability, unidentified direct targets, and an absence of rigorous clinical trials—that must be addressed in future research. Future research should focus on identifying how AST directly binds to cellular targets, developing better delivery systems, and conducting well-designed clinical studies to confirm its therapeutic value.

## Figures and Tables

**Table 1:** Summary of the Anti-Inflammatory Effects of AST Across Various Organs.

Organ	Experimental Model	Astaxanthin Dosage and Experimental Parameters in Preclinical Studies	Regulated Key Signaling Pathways	Autophagy Regulation Direction	Primary Effects	Ref.
Joint	ACLT-induced Osteoarthritis (OA) mouse model	NP@PolyRHAPM containing 50% AST; 5 μM (in vitro; cytotoxicity > 10 μM)	Inhibits the NLRP3 and ROS levels; promotes M1→M2 macrophage polarization	↑	Reduces ROS levels and enhances chondrocyte viability	[52]
Eye	Scopolamine-induced dry eye disease (DED) model	In vivo: 10 μM, 4× daily; in vitro: 10 μM & 25 μM, 6 h	Activates SLC7A11/GPX4 pathway	↑	Inhibits ferroptosis, alleviates DED	[54]
Eye	NaIO_3_-induced Age-Related Macular Degeneration (AMD) model	5, 10, 20 μmol/L (pre- or co-intervention)	Inhibits NLRP3 and LC3 expression	↓	Reduces NaIO_3_-induced photoreceptor inflammation and cell death	[32]
Liver	Acetaminophen (APAP)-induced liver injury	In vivo: 100 mg/kg (AST equivalent from HMSN@ASX: 10 mg/kg); in vitro: 25 μM & 50 μM	Activates Nrf2/HO-1; inhibits NF-κB pathway	↑	Attenuates hepatic tissue damage	[55]
Liver	70% hepatic IR injury	60 mg/kg	Inhibits ROS/MAPK pathway	↓	Reduces oxidative stress and improves liver function	[33]
Liver	BDL & CCl_4_-induced liver fibrosis	80 mg/kg	Inhibits TGF-β1/Smad/NF-κB pathway	↓	Suppresses excessive autophagy and alleviates fibrosis	[56]
Liver	ConA/TNF-α-induced autoimmune hepatitis	In vivo: 20 mg/kg (low), 40 mg/kg (high); in vitro: 80 μM	Inhibits phosphorylation of JNK, p38 MAPK, and ERK	↓	Improves liver function and reduces pathological injury	[38]
Stomach	*Helicobacter pylori* (*H. pylori*)-infected gastric epithelial cells	25 or 50 nM, 3 h pre-incubation	Activates AMPK; inhibits Akt/mTOR signaling	↑	Protects against *H. pylori*-induced cellular damage	[40]
Pancreas	Ceruletide-induced acute pancreatitis(AP) in mice	40 mg/kg	Activates Nrf2; downregulates Beclin-1 & Slc7a11	↑	Enhances autophagy in acinar cells and inhibits ferroptosis	[46]
Pancreas	Cerulein-induced AP in mice	40 mg/kg, i.p.	Inhibits JAK/STAT3 pathway	↓	Effectively ameliorates AP	[57]
Kidney	High-fat diet & STZ-induced diabetic nephropathy	In vivo: 25 mg/kg/day, oral, 3 weeks; in vitro: 2–25 μg/mL, 24 h	Upregulates LC3-II/I; downregulates VEGF-B, TGF-β2, p62, α-SMA	↑	Attenuates diabetic kidney injury	[31]
Kidney	Bilateral renal IR injury in rats	5, 10, 25 mg/kg/day	Increases Beclin-1 & LC3b; decreases p62	↑	Protects renal tissue	[30]
Testis	Testicular torsion-induced IR injury	1 mg/kg/day	Upregulates Beclin-1; slightly downregulates Caspase-3	↑	Reduces testicular damage via autophagy induction and apoptosis inhibition	[58]
Brain	Primary porcine brain capillary endothelial cells (PBCECs) modeling BBB	10 μM (16 h pre-incubation); 50 μM (co-incubation with LPS)	Increases LC3B-II; suppresses mTOR, p-mTOR, p-S6RP	↑	Protects against Alzheimer’s-related BBB dysfunction and neuroinflammation	[42]
Brain	Aβ_25–35_-induced Alzheimer’s disease (AD) cell model	Cellular: 25–50 μg/mL AST-DHA; Animal: 30 mg/kg/day AST-DHA & F-AST	Activates AMPK-ULK1; inhibits AKT-mTOR	↑	Reduces Aβ plaques and phosphorylated Tau protein	[41]
Brain	H_2_O_2_-induced oxidative damage in SH-SY5Y cells	80 μg/L, pre-incubation for 24 h	Activates Akt/mTOR pathway	↓	Inhibits excessive mitophagy and protects against neuronal damage	[44]

Note: This table summarizes the anti-inflammatory effects of AST mediated by autophagy regulation across various organs. In the “Autophagy Regulation Direction” column of the table, the “↓” arrow indicates a decrease in the levels of autophagy-related markers (such as LC3-II, Beclin1) or an increase in p62 accumulation, suggesting inhibition of autophagic activity; the “↑” arrow indicates an increase in the levels of such markers or a decrease in p62 accumulation, suggesting promotion of autophagic activity. Evidence from references [32,40,41,42,44,54] is derived from in vitro cell-based experiments, whereas the findings in references [30,31,37,38,46,52,55,56,57,58] are supported by in vivo studies.

## Data Availability

No new data were created or analyzed in this study.
